# Effect of K^+^ and Ca^2+^ on the indole-3-acetic acid- and fusicoccin-induced growth and membrane potential in maize coleoptile cells

**DOI:** 10.1093/aobpla/plv070

**Published:** 2015-06-30

**Authors:** Agnieszka Siemieniuk, Waldemar Karcz

**Affiliations:** Department of Plant Physiology, Faculty of Biology and Environmental Protection, University of Silesia, Jagiellońska 28, 40-032 Katowice, Silesia, Poland

**Keywords:** Auxin, calcium, coleoptile, elongation growth, fusicoccin, potassium, *Zea mays*

## Abstract

The role of potassium (K^+^) and calcium (Ca^2+^) in the regulation of plant growth and development is complex and needs a diverse range of physiological studies. Both elements are essential for satisfactory crop production. Here, the effects of K^+^ and Ca^2+^ ions on endogenous growth and growth in the presence of either indole-3-acetic acid (IAA) or fusicoccin (FC) were studied in maize (*Zea mays*) coleoptiles. Membrane potentials of coleoptile parenchymal cells, incubated in media containing IAA, FC and different concentrations of K^+^ and Ca^2+^, were also determined. Growth experiments have shown that in the absence of K^+^ in the incubation medium, both endogenous and IAA- or FC-induced growth were significantly inhibited by 0.1 and 1 mM Ca^2+^, respectively, while in the presence of 1 mM K^+^ they were inhibited only by 1 mM Ca^2+^. At 10 mM K^+^, endogenous growth and growth induced by either IAA or FC did not depend on Ca^2+^ concentration. TEA-Cl, a potassium channel blocker, added 1 h before IAA or FC, caused a reduction of growth by 59 or 45 %, respectively. In contrast to TEA-Cl, verapamil, the Ca^2+^ channel blocker, did not affect IAA- and FC-induced growth. It was also found that in parenchymal cells of maize coleoptile segments, membrane potential (*E*_m_) was strongly affected by the medium K^+^, independently of Ca^2+^. However, lack of Ca^2+^ in the incubation medium significantly reduced the IAA- and FC-induced membrane potential hyperpolarization. TEA-Cl applied to the control medium in the same way as in growth experiments caused *E*_m_ hyperpolarization synergistic with hyperpolarization produced by IAA or FC. Verapamil did not change either the *E*_m_ of parenchymal cells incubated in the control medium or the IAA- and FC-induced membrane hyperpolarization. The data presented here have been discussed considering the role of K^+^ uptake channels in regulation of plant cell growth.

## Abstract (precis)

The role of K^+^ and Ca^2+^ in plant growth and development is complex and needs extensive physiological studies. Here, the effects of K^+^ and Ca^2+^ ions on growth and membrane potential in the presence of either auxin (IAA) or fusicoccin (FC) were studied in maize coleoptiles. The results presented in this article demonstrate that the inhibitory effect of Ca^2+^ on growth is significantly restored in the presence of K^+^. This effect is probably due to competitive inhibition of K^+^ channels by Ca^2+^ ions, although other possibilities should be taken into consideration.

## Introduction

Potassium (K^+^) is the most abundant cation in plant tissues, constituting up to 10 % of plant dry weight (Leigh and Wyn Jones 1984; Epstein and Bloom 2005). The cytosolic K^+^ concentration, ranging from 60 to 200 mM, plays a role in such well-characterized functions as electrical compensation of anions, control of cell membrane potential, enzyme activation and long-distance transport of sucrose and nitrate (reviewed in Ahmad and Maathuis 2014; Anschütz *et al.* 2014; Véry *et al.* 2014, Zörb *et al.* 2014). Potassium is also involved in turgor-driven processes such as cell elongation, phototropism, gravitropism and stomatal movement (reviewed in Becker and Hedrich 2002). It is currently well established that auxin-induced growth in maize coleoptile segments involves K^+^ uptake through voltage-dependent, inwardly rectifying K^+^ channels (ZMK1, *Zea mays* K^+^ channel 1), the activity of which contributes to water uptake and consequently cell expansion (reviewed in Hager 2003). It has been shown that apart from posttranslational, auxin-dependent up-regulation of the K^+^ uptake channels, auxin also regulates the expression of the maize K^+^ uptake channel gene *ZMK1* (Philippar *et al.* 1999). Use of the patch-clamp technique on maize coleoptile protoplasts (Philippar *et al.* 1999; Thiel and Weise 1999) has confirmed some earlier studies which showed that auxin-induced growth strictly depends on external K^+^ supply (Claussen *et al.* 1997). The idea that the K^+^ uptake channels are crucial for auxin-induced growth comes also from experiments in which cell elongation and K^+^ conductance appeared to be sensitive to extracellular calcium (Ca^2+^) and tetraethylammonium (TEA), a K^+^ channel blocker (Thiel *et al.* 1996).

The divalent Ca^2+^ cation is an essential nutrient with diverse intra- and extracellular functions (Hofer and Brown 2003; Kudla *et al.* 2010). In plants, perception of most abiotic stresses results in the generation of calcium signals, which, in turn, elicit distinct Ca^2+^ concentration-dependent responses (McAinsh and Pittman 2009). Calcium, as one of the most important second messengers, is involved in a multitude of cellular processes, by which it exerts influence on nearly every aspect of plant growth and development (for review, see Vanneste and Friml 2009, 2013; Kudla *et al.* 2010; Schönknecht 2013).

To understand how auxin signals, particularly the ion transport-dependent ones, are transduced at a cellular level to elicit growth responses, we have performed experiments investigating the effects of K^+^ and Ca^2+^ on endogenous growth (growth without growth substances) and growth in the presence of either indole-3-acetic acid (IAA) or fusicoccin (FC) in maize coleoptile segments. In addition, membrane potential changes in parenchymal cells of coleoptile segments, incubated in the presence or absence of K^+^, Ca^2+^ and growth stimulators, were examined.

## Methods

### Plant material

Experiments were conducted on coleoptile segments obtained from 4-day-old seedlings grown in dark at 27 ± 1 °C. The 10-mm-long segments, with their first leaves removed, were cut from coleoptiles 3 mm below the tip. Conditions for growing the maize seedlings have been described previously (Karcz and Burdach 2002).

### Growth measurements

Growth measurements were performed with an angular position transducer (TWK Electronic, Düsseldorf, Germany) as described previously by Karcz and Burdach (2002) and by Polak *et al.* (2011). In the experiments, five unabraded coleoptile segments were strung on a stainless steel needle and inserted vertically in an intensively aerated medium (5 mL for each coleoptile segment), the composition of which varied dependently on the variant of the experiment (0.0, 1.0, 10.0 mM KCl; 0.0, 0.1, 1.0 mM CaCl_2_; 0.1 mM NaCl). In the growth measurement studies, every single experiment (extension of a stack of five segments) performed in a medium with a fixed ion concentration was repeated at least eight times. Temperature of growth solutions was thermostatically controlled at 25 ± 1 °C (LW 102, Auritronic, Poland).

Growth was sampled for 10 h at regular 3-min intervals by the CX 721 converter (Elmetron, Poland). Growth substances, IAA or FC, were administered after 2 h of the experiment. All data are expressed as growth rate (µm s^−1^ cm^−1^) and coleoptile elongation (µm cm^−1^).

### Electrophysiological measurements

Electrophysiological experiments were carried out on intact 10-mm-long maize coleoptile segments prepared as for growth experiments. The experimental technique previously described by Karcz and Burdach (2002), Kurtyka *et al.* (2011) and Polak *et al.* (2012) was used. Membrane potential (*E*_m_) was measured by recording the voltage between a 3 M KCl-filled glass micropipette inserted into the parenchymal cells and a reference electrode in the bathing medium. Before the electrophysiological experiments, maize coleoptile segments were preincubated for 110 min in an intensively aerated solution of the same composition as for growth measurements. After preincubation, a single segment was transferred into a perfusion electrophysiological chamber filled with a bathing medium of identical ion composition as the preincubation medium. Subsequently, a microelectrode was inserted into the parenchymal cell by means of a micromanipulator (Hugo Sachs Electronik, March-Hugstteten, Germany). After stabilization of the *E*_m_ (<10 min), the bathing medium was exchanged with a solution of the same ion composition, containing, in addition, IAA or FC. Each single electrophysiological experiment means measurement of the *E*_m_ in coleoptile parenchymal cell (one cell per one segment), repeated at least seven times. A peristaltic pump (Type Peri-Star PRO; World Precision Instruments, Sarasota, FL, USA) was used for changing the bathing medium in the chamber (usually 4-fold within <2 min). Micropipettes were pulled on a vertical pipette puller (Model L/M-3P-A; List-Medical, Germany) from borosilicate glass capillaries (Type 1B150F-3; World Precision Instruments). Tip diameter did not exceed 1 µm.

### Chemicals

Indole-3-acetic acid (IAA) (Merck, Germany) was used as potassium salt, since it could be rapidly dissolved in water. Indole-3-acetic acid was used at 10 µM. Fusicoccin (Sigma, USA) was dissolved in ethanol and added to the incubation medium at a final concentration of 1 µM. The maximal ethanol concentration of 0.2 % did not affect the growth of coleoptile segments (data not shown). Tetraethylammonium chloride (Sigma) and verapamil (Sigma) were dissolved in deionized water and used at a final concentration of 30 mM and 50 µM, respectively. Stock solutions of TEA and verapamil were prepared in concentrations 100-fold greater than those used in experiments.

### Data analysis

Data were analysed with Statistica software for Windows (StatSoft 2008, STATISTICA data analysis software system, version 8.0, http://www.statsoft.com, USA) at a significance level 0.05.

One-way ANOVA was used to test statistical differences in coleoptile growth among the considered groups (that is, various calcium concentrations with fixed potassium level and various channel blockers). Data were tested for normal distribution and variance homogeneity using Levene's test. Subsequently, the post hoc least significant difference (LSD) test was used for further analysis (*P* < 0.05). Student's *t*-test was used to estimate the significance of differences between membrane potential values recorded at 0 and 30 min of the measurements. The curves of membrane potential changes were fitted with least-squares linear regression and subsequently smoothed using Statistica software for Windows (Version 8.0).

## Results

### Effect of K^+^ and Ca^2+^ on endogenous growth

**Figure 1.**
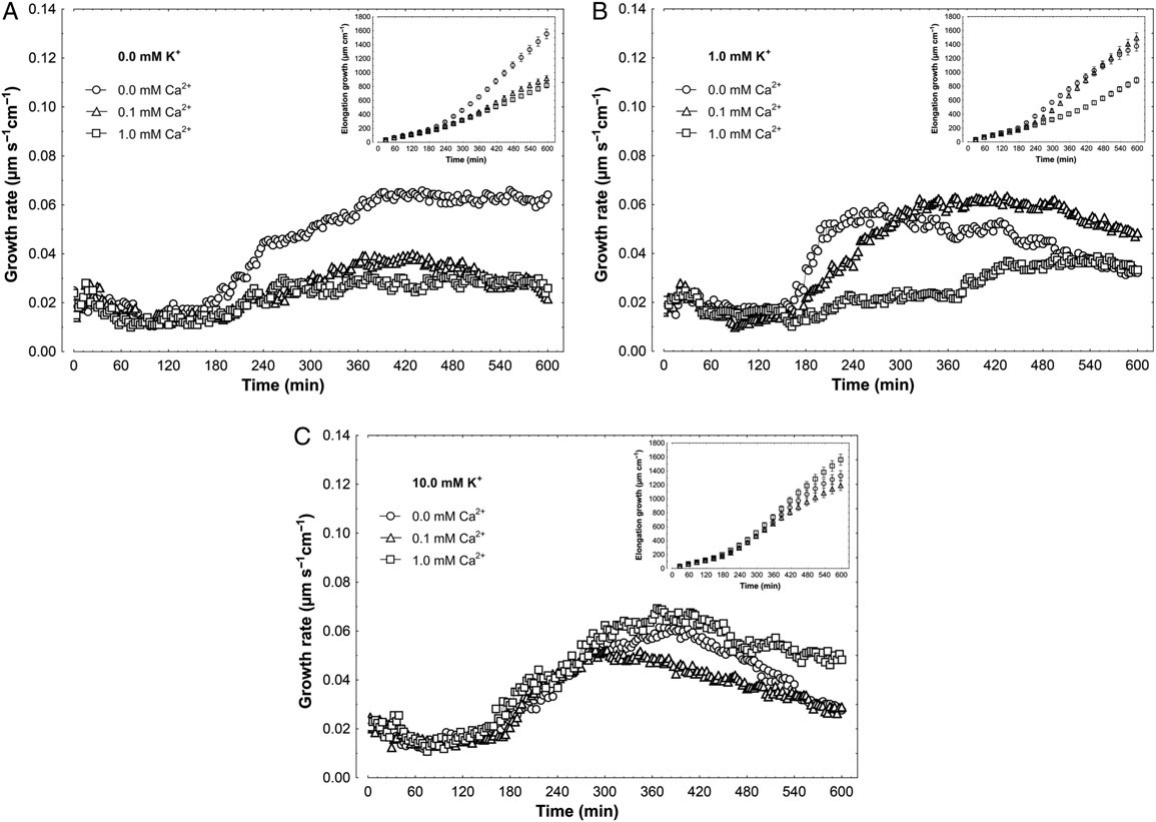
Effect of K^+^ and Ca^2+^ ions on the endogenous growth rate (growth without growth substances) of maize coleoptile segments. (A) Growth rate (µm s^−1^ cm^−1^) of coleoptile segments incubated in the medium with 0.0 mM K^+^ and Ca^2+^ at 0.0, 0.1 and 1.0 mM. (B) As in A but with 1.0 mM K^+^. (C) As in A and B but with 10 mM K^+^ in the medium. The growth rate of five coleoptile segments (10 mm in length) was recorded for 10 h in an intensively aerated incubation medium (5 mL segment^−1^) by means of an angular position transducer (TWK Electronic). Inset shows total elongation growth calculated as the sum of extensions measured in 3-min intervals for 10 h. All curves are mean values of at least eight independent measurements. Bars indicate ±SE.

The effects of K^+^ (0.0, 1.0 and 10 mM) and Ca^2+^ (0.0, 0.1 and 1 mM) on the growth rate of maize coleoptile segments incubated in control medium (endogenous growth) are shown in Fig. 1. On the basis of growth rate responses, the total elongation growth (calculated as the sum of extensions from 3-min intervals) of coleoptile segments was also obtained (Fig. 1, insets). Taking these data into account, it should be stated that in media without K^+^, Ca^2+^ at 0.1 and 1 mM significantly (*F*_2,23_ = 15.21, *P* < 0.01) decreased endogenous growth of coleoptile segments by ∼50 %, as compared with growth in medium without Ca^2+^ and K^+^ (1554.4 ± 140.4 µm cm^−1^; mean ± SE, *n* = 8, Fig. 1A, inset). In the presence of 1 mM K^+^, endogenous growth of coleoptile segments was diminished only at 1 mM Ca^2+^ (*F*_2,24_ = 11.17, *P* < 0.01) (Fig. 1B). At 10 mM K^+^, endogenous growth of segments only slightly (especially over the first 6 h) depended on Ca^2+^ concentration (*F*_2,24_ = 3.47, *P* = 0.047) (Fig. 1C). Interestingly, in the absence of Ca^2+^ in the medium, elongation growth of coleoptile segments was comparable at all K^+^ concentrations studied (*F*_2,25_ = 1.25, *P* = 0.302) (Fig. 1).

### Effect of K^+^ and Ca^2+^ on IAA- and FC-induced growth

**Figure 2.**
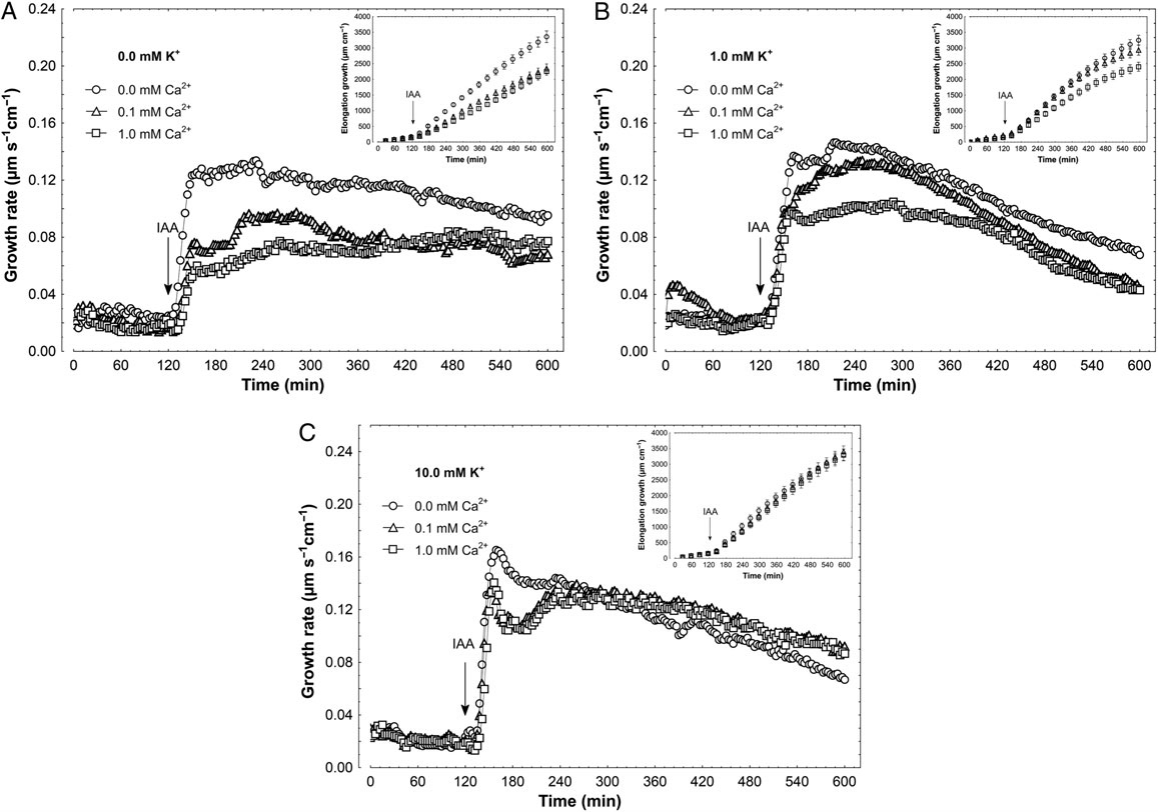
Effect of K^+^ and Ca^2+^ ions on the IAA-induced growth rate of maize coleoptile segments. (A) IAA-induced growth rate (µm s^−1^ cm^−1^) of coleoptile segments incubated in the medium with 0.0 mM K^+^ and Ca^2+^ at 0.0, 0.1 and 1.0 mM. (B) As in A but with 1.0 mM K^+^. (C) As in A and B but with 10 mM K^+^ in the medium. The growth rate of five coleoptile segments (10 mm in length) was recorded for 10 h in an intensively aerated incubation medium (5 mL segment^−1^) by means of an angular position transducer (TWK Electronic). Coleoptile segments were first preincubated for 2 h in a control medium, whereupon IAA was added at a final concentration of 10 µM. Inset shows total elongation growth, calculated as the sum of extensions measured in 3-min intervals for 10 h. All curves are mean values of at least eight independent measurements. Bars indicate ±SE.

**Figure 3.**
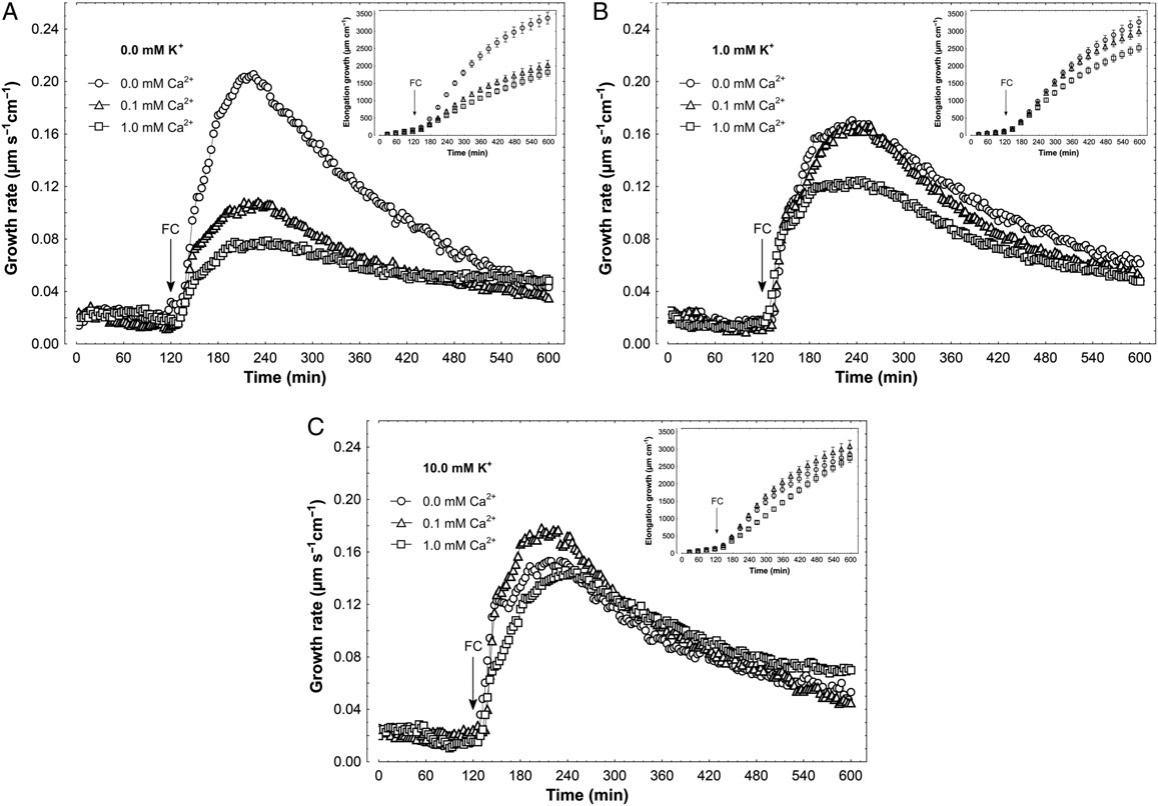
Effect of K^+^ and Ca^2+^ ions on the FC-induced growth rate of maize coleoptile segments. (A) FC-induced growth rate (µm s^−1^ cm^−1^) of coleoptile segments incubated in the medium with 0.0 mM K^+^ and Ca^2+^ at 0.0, 0.1 and 1.0 mM. (B) As in A but with 1.0 mM K^+^. (C) As in A and B but with 10 mM K^+^ in the medium. The growth rate of five coleoptile segments (10 mm in length) was recorded for 10 h in an intensively aerated incubation medium (5 mL segment^−1^) by means of an angular position transducer (TWK Electronic). Coleoptile segments were first preincubated for 2 h in a control medium, whereupon FC was added, at a final concentration of 1 µM. Inset shows total elongation growth calculated as the sum of extensions measured in 3-min intervals for 10 h. All curves are mean values of at least eight independent measurements. Bars indicate ±SE.

When IAA, at a final concentration of 10 µM, was added to the control medium, 2 h after the start of the experiment, a strong increase was observed in the growth rate (Fig. 2). The kinetics of IAA-induced growth could be divided into two phases (biphasic reaction) as previously described (Vanderhoef and Stahl 1975; Kazama and Katsumi 1976; Vanderhoef and Dute 1981; Philippar *et al*. 1999; Polak 2010); the first phase, very rapid, was followed by a long-lasting one, which began ∼30 min after auxin addition. In the absence of K^+^ in the incubation medium (Fig. 2A), Ca^2+^ at 0.1 and 1 mM significantly (*F*_2,22_ = 4.83, *P* = 0.018) diminished IAA-induced elongation growth by 31 %, as compared with IAA-induced growth in medium without Ca^2+^ and K^+^ (3357.8 ± 372.5 µm cm^−1^; mean ± SE, *n* = 9, Fig. 2A, inset). However, in the presence of 1 mM K^+^, IAA-induced elongation growth of coleoptile segments was significantly reduced (by 26 %) at 1 mM Ca^2+^ (*F*_2,25_ = 3.757, *P* = 0.037). When IAA was added to the medium containing 10 mM K^+^, auxin-induced growth did not depend on Ca^2+^ concentration (*F*_2,21_ = 0.031, *P* = 0.97) (Fig. 2C). Effects of K^+^ and Ca^2+^ were also studied for the growth of maize coleoptile segments incubated in the presence of FC (Fig. 3). This fungal toxin is known to enhance H^+^-ATPase activity, through phosphorylation of the penultimate Thr, as well as induce elongation growth (Marrè 1979; Kinoshita and Shimazaki 2001). Fusicoccin added to the incubation medium in the same way as IAA, at a final concentration of 1 µM, enhanced the endogenous growth of maize coleoptile segments to a level comparable with growth seen in the presence of IAA (Fig. 2). Independently of the ionic composition of the medium, FC added at 2 h of segment preincubation caused rapid growth with bell-shaped kinetics (Fig. 3). In the absence of K^+^ in incubation medium (Fig. 3A), Ca^2+^ at 0.1 and 1 mM significantly inhibited FC-induced elongation growth (*F*_2,23_ = 12.79, *P* < 0.01) by 46 % compared with growth in medium without Ca^2+^ and K^+^ (3378.3 ± 338.6 µm cm^−1^; mean ± SE, *n* = 8, Fig. 3A, inset). In medium with 1 mM K^+^ and Ca^2^,^+^ at both concentrations, inhibition of FC-induced growth was significant (*F*_2,25_ = 4.07, *P* = 0.029) but did not exceed 25 % (Fig. 3B). As in the case of IAA, FC-induced growth in a medium containing 10 mM K^+^ did not depend on Ca^2+^ concentration (*F*_2,24_ = 0.215, *P* = 0.807) (Fig. 3C).

### Effect of K^+^ and Ca^2+^ channel blockers on IAA- and FC-induced growth

**Figure 4.**
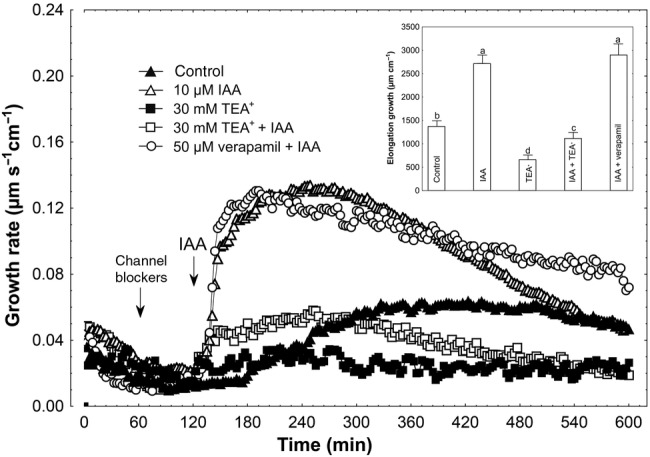
Effect of K^+^ and Ca^2+^ channel blockers (TEA-Cl and verapamil, respectively) on the IAA-induced growth rate (μm s^−1^ cm^−1^) of maize coleoptile segments. Coleoptile segments were first preincubated for over 1 h in a control medium, whereupon channel blockers were added. At 2 h, IAA was added to the incubation medium, at a final concentration of 10 µM. Inset on the right shows the total elongation growth, presented in a bar graph, calculated as the sum of extensions between 120 and 600 min of the experiment. Values are the mean of at least nine independent experiments. Bars indicate mean ± SE. Mean values followed by the same letter are not significantly different from each other according to the LSD test (*P* < 0.05).

**Figure 5.**
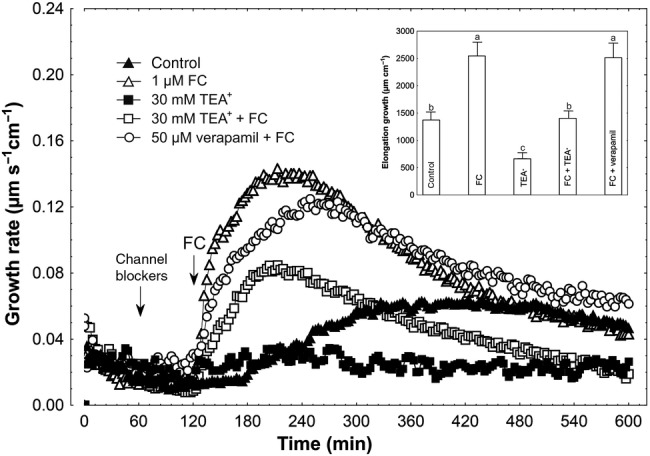
Effect of K^+^ and Ca^2+^ channel blockers (TEA-Cl and verapamil, respectively) on the FC-induced growth rate (μm s^−1^ cm^−1^) of maize coleoptile segments. Coleoptile segments were first preincubated for over 1 h in a control medium, whereupon channel blockers were added. At 2 h, FC was added to the incubation medium, at a final concentration of 1 µM. Inset on the right shows the total elongation growth, presented in a bar graph, calculated as the sum of extensions between 120 and 600 min of the experiment. Values are the mean of at least nine independent experiments. Bars indicate mean ± SE. Mean values followed by the same letter are not significantly different from each other according to the LSD test (*P* < 0.05).

Figure 4 shows the effects of K^+^ and Ca^2+^ channel blockers (TEA-Cl and verapamil, respectively) on IAA-induced growth of maize coleoptile segments incubated in the presence of 1 mM K^+^ and 0.1 mM Ca^2+^. The segments were first preincubated for 1 h before the blockers were added. At 2 h, IAA was applied to the incubation medium. Data in Fig. 4 indicate that TEA-Cl, added at a final concentration of 30 mM, significantly decreased the growth of IAA-incubated coleoptile segments (*F*_1,17_ = 46.61, *P* < 0.01) (2935.3 ± 324.6 µm cm^−1^; mean ± SE, *n* = 9) by 59 %. TEA-Cl added alone 1 h after start of the experiment diminished growth (*F*_1,16_ = 24.18, *P* < 0.01) by 40 % as compared with control (1494.7 ± 146.4 µm cm^−1^; mean ± SE, *n* = 9) (Fig. 4, inset). Addition of verapamil, at a final concentration of 50 µM, in the same time protocol as TEA-Cl, increased IAA-induced growth by 7 % (statistically not significant, *F*_1,16_ = 0.01, *P* = 0.76) (Fig. 4, inset). As presented in Fig. 5, FC added to incubation medium containing TEA-Cl enhanced the growth of coleoptile segments (*F*_1,18_ = 39.81, *P* < 0.01) to a level 45 % lower than FC alone (2998.6 ± 265.0 µm cm^−1^; mean ± SE, *n* = 10). Verapamil added in the same way as TEA-Cl did not change elongation growth in response to FC (*F*_1,17_ = 1.52, *P* = 0.23).

### Effect of K^+^ and Ca^2+^ on the IAA- and FC-induced *E*_m_ changes

**Figure 6.**
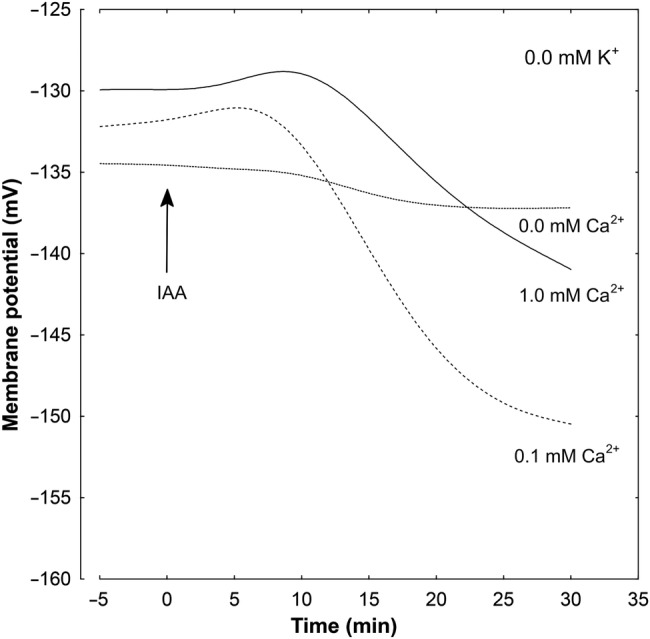
Effect of different concentrations of Ca^2+^ ions on the IAA-induced changes in the *E*_m_ measured in maize coleoptile parenchymal cells in a medium without K^+^. Coleoptile segments were incubated in the indicated medium (the same as in growth experiments) for 110 min; afterwards a single segment was transferred into an electrophysiological chamber containing the same medium. Measurements of membrane potential (30 min) were carried out after insertion of a microelectrode into the cell (one cell per one segment) and stabilization of the *E*_m_ (<10 min) at 2 h (0 min). Indole-3-acetic acid, at a final concentration of 10 µM, was added after 2 h of segment preincubation in the indicated medium; for the last 10 min, coleoptile segments were incubated in the electrophysiological chamber. Representative curves are shown. These curves were fitted with least-squares linear regression and subsequently smoothed using Statistica software for Windows (Version 8.0).

**Figure 7.**
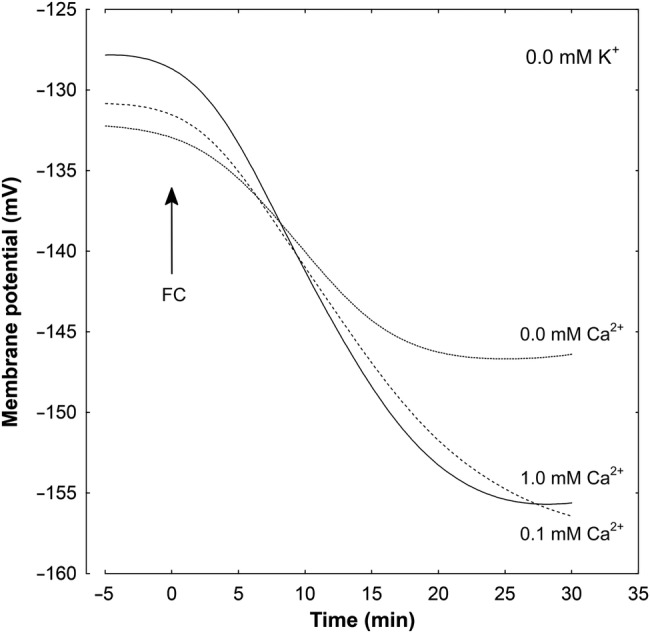
Effect of different concentrations of Ca^2+^ ions on the FC-induced changes in the *E*_m_ measured in maize coleoptile parenchymal cells in a medium without K^+^. Coleoptile segments were incubated in the indicated medium (the same as in growth experiments) for 110 min; afterwards a single segment was transferred into an electrophysiological chamber containing the same medium. Measurements of membrane potential (30 min) were carried out after insertion of a microelectrode into the cell (one cell per one segment) and stabilization of the *E*_m_ (<10 min) at 2 h (0 min). Fusicoccin, at a final concentration of 1 µM, was added after 2 h of segment preincubation in the indicated medium; for the last 10 min, coleoptile segments were incubated in the electrophysiological chamber. Representative curves are shown. These curves were fitted with least-squares linear regression and subsequently smoothed using Statistica software for Windows (Version 8.0).

Results shown in Table 1 indicate that the membrane potential of parenchymal cells of maize coleoptile segments depended on K^+^ concentration, not on Ca^2+^ concentration (Table 1, column A). For example, a 10-fold increase in K^+^ concentration, independently of Ca^2+^ concentration, caused depolarization by ∼56 mV, which is near the value predicted from the Nernst equation. Addition of IAA to the incubation medium (Table 1, Fig. 6) caused transient depolarization of the *E*_m_, followed by a delayed hyperpolarization, during which membrane potential became more negative than the original potential. In the presence of IAA, membrane hyperpolarization was not statistically significant in media without Ca^2+^ (Table 1, Fig. 6). In contrast to IAA, FC caused an immediate hyperpolarization of the *E*_m_, attaining significantly higher values independently of K^+^ and Ca^2+^ concentrations. Similarly to IAA, the FC-induced membrane potential hyperpolarization was inhibited in the absence of Ca^2+^ (Table 1, Fig. 7) and showed a decrease with increasing K^+^ concentration.

**Table 1.** Membrane potential (*E*_m_, mV) in parenchymal coleoptile cells. Data (mean ± SE) are mean values of at least seven independent experiments. Coleoptile segments were incubated in the indicated medium (the same as in growth experiments) for 110 min; afterwards a single segment was transferred into an electrophysiological chamber containing the same medium. Measurements of membrane potential (30 min) were carried out after insertion of a microelectrode into the cell and stabilization of the *E*_m_ (<10 min) at 2 h (0 min, column A). Indole-3-acetic acid and FC were added after 2 h of segment incubation in the indicated medium; for the last 10 min before IAA or FC addition, coleoptile segments were incubated in the electrophysiological chamber. ns, Not statistically significant. *Statistically significant (*P* < 0.05).

Treatment			Membrane potential (mV)					
	K^+^	Ca^2+^	A 0 min	Transient depolarization	B 10 min	C 20 min	D 30 min	Δ*E*_m_ (D − A) (mV)
	(mM)							
IAA	0.0	0.0	−138.6 ± 7.1	1.4	−138.2 ± 6.5	−141.1 ± 7.6	−141.2 ± 7.7	−2.6^ns^
		0.1	−136.5 ± 6.5	6.0	−136.2 ± 7.3	−151.5 ± 6.9	−153.7 ± 6.8	−17.2*
		1.0	−134.1 ± 6.5	5.3	−130.7 ± 6.1	−140.8 ± 7.4	−144.2 ± 7.0	−10.1*
IAA	1.0	0.0	−124.5 ± 5.9	1.8	−124.0 ± 6.2	−126.5 ± 7.0	−127.1 ± 6.8	−2.6^ns^
		0.1	−120.7 ± 6.1	5.5	−118.3 ± 5.8	−126.8 ± 6.7	−129.3 ± 7.1	−8.6*
		1.0	−122.4 ± 6.3	7.3	−116.3 ± 4.9	−129.0 ± 7.2	−132.4 ± 6.5	−10.0*
IAA	10	0.0	−64.7 ± 5.0	1.5	−64.5 ± 5.4	−67.1 ± 3.9	−67.4 ± 5.1	−2.7^ns^
		0.1	−65.6 ± 4.3	2.8	−63.2 ± 5.0	−69.6 ± 5.2	−71.0 ± 4.7	−5.4*
		1.0	−64.8 ± 4.1	2.2	−64.0 ± 4.7	−70.3 ± 5.1	−71.3 ± 4.3	−6.5*
FC	0.0	0.0	−132.7 ± 7.1	–	−139.0 ± 6.3	−146.5 ± 6.9	−146.6 ± 7.1	−13.9*
		0.1	−131.2 ± 6.6	–	−140.8 ± 7.1	−152.2 ± 7.3	−155.8 ± 7.6	−24.6*
		1.0	−128.0 ± 7.2	–	−142.1 ± 6.9	−153.5 ± 7.9	−155.4 ± 7.9	−27.4*
FC	1.0	0.0	−120.1 ± 5.8	–	−125.5 ± 6.2	−128.1 ± 6.5	−128.0 ± 6.1	−7.9*
		0.1	−118.7 ± 5.5	–	−126.7 ± 6.6	−135.8 ± 6.4	−138.4 ± 7.2	−19.7*
		1.0	−121.3 ± 6.1	–	−128.0 ± 6.4	−135.0 ± 7.2	−135.9 ± 6.3	−14.6*
FC	10	0.0	−65.5 ± 4.2	–	−66.9 ± 3.9	−69.2 ± 5.1	−69.5 ± 4.1	−4.0^ns^
		0.1	−68.4 ± 4.7	–	−75.2 ± 4.6	−78.5 ± 4.8	−81.3 ± 5.3	−12.9*
		1.0	−64.6 ± 4.6	–	−71.0 ± 5.1	−75.7 ± 4.9	−77.3 ± 4.8	−12.7*

### Effect of TEA-Cl and verapamil on the IAA- and FC-induced *E*_m_ changes

**Figure 8.**
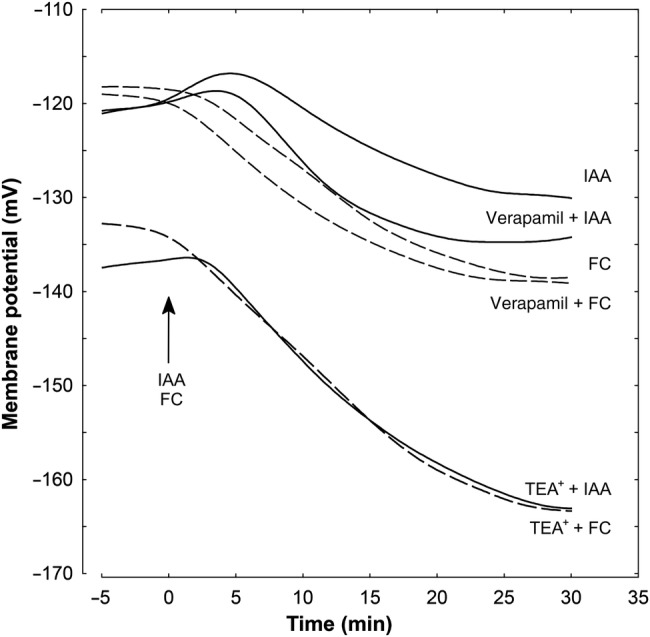
Effect of K^+^ and Ca^2+^ channel blockers (TEA-Cl and verapamil, respectively) on the IAA- and FC-induced changes in the *E*_m_ measured in maize coleoptile parenchymal cells in a medium with 1 mM K^+^ and 0.1 mM Ca^2+^. Coleoptile segments were incubated in the indicated medium (the same as in growth experiments) for 60 min; afterwards the channel blockers were added. A total of 110 min after the start of preincubation, a single segment was transferred into an electrophysiological chamber containing the same medium (with channel blockers). Measurements of membrane potential (30 min) were carried out after insertion of a microelectrode into the cell (one cell per one segment) and stabilization of the *E*_m_ (<10 min) at 2 h (0 min). Indole-3-acetic acid or FC, at a final concentration of 10 and 1 µM, respectively, were added after 2 h of segment preincubation in the indicated medium; for the last 10 min, coleoptile segments were incubated in the electrophysiological chamber. Representative curves are shown. These curves were fitted with least-squares linear regression and subsequently smoothed using Statistica software for Windows (Version 8.0).

Figure 8 and Table 2 show the effects of TEA-Cl and verapamil on either IAA- or FC-induced *E*_m_ changes in parenchymal cells of maize coleoptile segments incubated in medium with 1 mM K^+^ and 0.1 mM Ca^2+^. Indole-3-acetic acid, added at 2 h of segment incubation in medium with 1 mM K^+^ and 0.1 mM Ca^2+^ (for the last 10 min, coleoptile segments were incubated in the electrophysiological chamber), caused transient depolarization of the *E*_m_ followed by a delayed membrane hyperpolarization during which membrane potential became by 9.2 mV more negative than the original potential (−121.0 ± 7.2 mV, mean ± SE, *n* = 13) (Fig. 8, Table 2). When TEA-Cl, at a final concentration of 30 mM, was added to the medium with 1 mM K^+^ and 0.1 mM Ca^2+^, after 1 h of segment incubation (for the last 10 min, coleoptile segments were incubated in the electrophysiological chamber), the *E*_m_ of cells was hyperpolarized by 16.3 mV. In turn, addition of IAA at this hyperpolarized state of membrane caused additional hyperpolarization of the *E*_m_ by 25.4 mV at 30 min. Verapamil, added to medium in the same way as TEA-Cl, did not affect the original *E*_m_ (Fig. 8, Table 2). Administration of IAA in the presence of verapamil induced hyperpolarization by 4.3 mV higher than for auxin alone. Similar dependences were recorded in the presence of FC and both channel blockers (Fig. 8, Table 2). All substances used with FC were added to the incubation medium in the same time protocol as was IAA. Addition of FC produced immediate hyperpolarization of *E*_m_, where the *E*_m_ was by 19.7 mV more negative than the original value (−121.6 ± 6.1 mV, mean ± SE, *n* = 9). At 30 mM TEA-Cl, FC-induced membrane hyperpolarization was by 30.3 mV more negative than the *E*_m_ value after 1 h of segment incubation in medium with TEA-Cl. Verapamil did not basically change the original *E*_m_ (Table 2, column A). Fusicoccin, added at 2 h to the incubation medium with verapamil, after 30 min, caused a 20.4 mV hyperpolarization (from −119.0 ± 4.5 to −139.4 ± 6.1 mV, Table 2).

**Table 2.** Membrane potential (*E*_m_, mV) in parenchymal coleoptile cells. Data (mean ± SE) are mean values of at least seven independent experiments. Coleoptile segments were incubated in the indicated medium (the same as in growth experiments) for 110 min; afterwards a single segment was transferred into an electrophysiological chamber containing the same medium. Measurements of membrane potential (30 min) were carried out after insertion of a microelectrode into the cell and stabilization of the *E*_m_ (<10 min) at 2 h (0 min, column A). Indole-3-acetic acid or FC were added after 2 h of segment preincubation in medium with 1 mM KCl and 0.1 mM CaCl_2_; for the last 10 min, coleoptile segments were incubated in the electrophysiological chamber. TEA-Cl or verapamil was added after 1 h of segment preincubation. All values in last column are statistically significant (*P* < 0.05).

Treatment		Membrane potential (mV)					
		A 0 min	Transient depolarization	B 10 min	C 20 min	D 30 min	Δ*E*_m_ (D − A) (mV)
IAA	Control	−121.0 ± 7.2	5.2	−120.6 ± 5.9	−128.1 ± 7.1	−130.2 ± 7.5	9.2
	TEA^+^	−137.3 ± 6.9	2.6	−147.3 ± 6.5	−158.1 ± 6.9	−162.7 ± 7.7	25.4
	Verapamil	−120.7 ± 6.5	3.6	−126.5 ± 5.9	−134.1 ± 7.1	−134.2 ± 7.3	13.5
FC	Control	−121.6 ± 6.1	–	−126.7 ± 5.9	−135.7 ± 6.4	−141.3 ± 6.6	19.7
	TEA^+^	−133.0 ± 6.6	–	−146.6 ± 7.1	−159.4 ± 7.3	−163.3 ± 7.5	30.3
	Verapamil	−119.0 ± 4.5	–	−131.0 ± 5.5	−137.3 ± 5.8	−139.4 ± 6.1	20.4

## Discussion

The aim of this work was to determine the role of K^+^ and calcium ions in auxin- and FC-induced growth and *E*_m_ changes in maize (*Zea mays*) coleoptile cells. It is now well established that the PM H^+^-ATPase, which generates the electrochemical gradient of H^+^ providing the driving force for a broad range of secondary transporters, plays a key role in the regulation of plant growth and development. According to the so-called ‘acid growth theory’, auxin activates the PM H^+^-ATPase, which acidifies the apoplasm and causes activation of enzymes involved in cell wall loosening (for review, see Rayle and Cleland 1992; Lüthen *et al*. 1999; Hager 2003). At least in maize coleoptile segments, auxin-induced medium acidification is mediated by the activity and/or amount of PM H^+^-ATPase (Hager *et al.* 1991; Frias *et al.* 1996). Interestingly, a similar scenario to that of the auxin-induced activation of PM H^+^-ATPase has been also reported for K^+^ uptake channels (ZMK1), as mentioned in the Introduction. Fusicoccin, a phytotoxic terpenoid produced by the fungus *Fusicoccum amygdali* (Ballio *et al.* 1964), mimics the effects of auxin in many respects (Marrè 1979), although its mode of action at the molecular level differs from that of auxin. It has been well documented that FC binds to the H^+^-ATPase/14-3-3 complex and stabilizes it, thus causing an increase in proton pump activity (Jahn *et al.* 1997; Baunsgaard *et al.* 1998; Fuglsang *et al.* 1999; Oecking and Hagemann 1999, Würtele *et al.* 2003). The data presented herein, showing that both IAA and FC result in acceleration of elongation growth as compared with endogenous growth (Figs 1–3) and that both substances cause hyperpolarization of the PM, that in case of IAA is preceded by a transient depolarization of the *E*_m_, are in good agreement with results obtained by other investigators (Cleland *et al.* 1977; Kutschera and Schopfer 1985*a*, *b*; Felle *et al.* 1986; Senn and Goldsmith 1988; Keller and Van Volkenburgh 1996; Karcz and Burdach 2002, 2007; Kurtyka *et al.* 2011; Rudnicka *et al.* 2014).

Growth experiments performed in media lacking K^+^ and Ca^2+^ revealed strong endogenous growth and growth in the presence of either auxin (IAA) or FC. This K^+^-independent growth was significantly diminished by 0.1 and 1.0 mM Ca^2+^ (Figs 1–3). When 1 mM K^+^ was included in the incubation medium, both IAA- and FC-induced growth of coleoptile segments were strongly restored, particularly at the lower Ca^2+^ concentration. However, in contrast to growth stimulated by IAA and FC, endogenous growth of coleoptile segments was still inhibited at 1 mM Ca^2+^. In medium containing 10 mM K^+^, endogenous growth and both IAA- and FC-induced growth depended weakly on Ca^2+^ and were comparable to those recorded in a medium without K^+^ and Ca^2+^. Both observations concerning growth, namely, its restoration at 1 mM K^+^ and its weak dependency on Ca^2+^ at 10 mM K^+^, may suggest that K^+^ channels are competitively inhibited by Ca^2+^, as previously proposed by Thiel *et al.* (1996). Another possibility, at least in the case of auxin action, is that it induces a rapid and transient increase in cytosolic Ca^2+^ concentration (Felle 1988; Gehring *et al.* 1990; Shishova and Lindberg 2004, 2010; Monshausen *et al.* 2011; Kirpichnikova *et al.* 2014) which, in turn, could inhibit PM H^+^-ATPase activity (Kinoshita *et al.* 1995; Polevoi *et al.* 1996; Brault *et al.* 2004; Fuglsang *et al.* 2007). Our results with TEA showed that when this K^+^-channel blocker was applied 1 h before addition of either IAA or FC, a 59 or 45 % growth reduction was observed, respectively (Figs 4 and 5). The lower inhibition of FC-induced growth by TEA is probably due to the fact that FC is unable to induce ZMK1 expression (Philippar *et al.* 1999). Inhibition of IAA- and FC-induced growth of maize coleoptile segments by TEA, used at the same concentration as in our experiments, was also shown previously by Claussen *et al.* (1997) and Tode and Lüthen (2001) and also recently by Burdach *et al.* (2014). Moreover, the authors found that TEA inhibits IAA-induced proton extrusion. In contrast to the K^+^-channel blocker, application of a Ca^2+^-channel blocker (verapamil) practically did not affect IAA- and FC-induced growth of maize coleoptile segments (Figs 4 and 5).

The above-mentioned results are generally in agreement with data obtained by Claussen *et al.* (1997) and Tode and Lüthen (2001), who showed that IAA- and FC-induced growth may occur in a K^+^-free medium lacking calcium. To explain the K^+^-independent growth of maize coleoptile segments incubated in a medium lacking K^+^ and Ca^2+^, Claussen *et al.* (1997) and Tode and Lüthen (2001) proposed a hypothesis assuming that the apoplastic Donnan space, being a huge reservoir for K^+^, can potentially supply this ion in amounts sufficient to maintain growth over a long time. If K^+^ in the Donnan space was replaced by Ca^2+^, the IAA- and FC-induced growth was abolished, indicating that in both cases K^+^ uptake was necessary for growth (Claussen *et al.* 1997; Tode and Lüthen 2001). Interestingly, IAA- and FC-pretreated coleoptile segments, which did not grow in the absence of K^+^ ions, displayed rapid growth if KCl was added to the medium (Claussen *et al.* 1997; Tode and Lüthen 2001). It is possible that the ZMK1 channel, as an orthologue of the *Arabidopsis* AKT1 channel (Geiger *et al.* 2009), may mediate the uptake of K^+^ under K^+^-limiting conditions. It is noteworthy that stimulation of coleoptile elongation growth in the presence of K^+^ and growth inhibition under the influence of Ca^2+^ were discovered in the 1950s (Cooil and Bonner 1957; Tagawa and Bonner 1957; Cleland and Rayle 1977).

Electrophysiological experiments showed that in parenchymal cells of coleoptile segments, membrane potential was strongly related to the medium K^+^ (Table 1, column A). This dependence did not change when calcium was included in the medium, suggesting that Ca^2+^ at 0.1 and 1.0 mM had no effect on K^+^ conductance. The above-described results are in line with data obtained in patch-clamp experiments by Thiel *et al.* (1996), who reported inhibition of K^+^ uptake channels at a higher (10 mM) concentration of Ca^2+^ in maize coleoptile protoplasts. Addition of IAA or FC to the incubation medium caused hyperpolarization of the *E*_m_, attaining higher values in the presence of FC (Table 1, Figs 6 and 7). There is no doubt that plasma membrane hyperpolarization in the presence of IAA or FC is a consequence of stimulated proton extrusion through the PM H^+^-ATPase (Rück *et al.* 1993; Hedrich *et al.* 1995; Hager 2003). In a medium lacking Ca^2+^, IAA- and FC-induced membrane hyperpolarization was strongly inhibited (Table 1, column Δ*E*_m_), although either IAA- or FC-induced growth was strongly stimulated (Figs 2 and 3). It is possible that under such conditions the stimulating effect of IAA and FC on K^+^ uptake and then on elongation growth does not require any additional activation of K^+^ uptake channels; they are probably already active enough.

Tetraethylammonium chloride applied after 1 h of segment incubation in the control medium produced hyperpolarization of the *E*_m_, which became by 16.3 mV more negative than the original membrane potential in control medium (−121.0 ± 7.2 mV) (Table 2). Indole-3-acetic acid added at 2 h to the medium containing TEA induced additional membrane hyperpolarization by 25.4 mV (Table 2), suggesting that both processes are synergistic. A similar effect was observed for FC, which hyperpolarized the *E*_m_ synergically with TEA (Table 2). In contrast to the K^+^-channel blocker, verapamil administered in the same time protocol as TEA did not change the *E*_m_ in parenchymal coleoptile cells incubated in the control medium (Table 2, column A). Indole-3-acetic acid or FC added to the medium with verapamil induced hyperpolarization of the *E*_m_ comparable to the one observed in the presence of IAA or FC alone.

## Conclusions

The results presented in this article demonstrate that the inhibitory effect of Ca^2+^ on endogenous growth and both IAA- and FC-induced growth is significantly restored in the presence of K^+^. This effect is probably due to the competitive inhibition of K^+^ channels by Ca^2+^ ions, although other possibilities could be taken into consideration.

## Sources of Funding

Founding was provided by University of Silesia.

## Contributions by the Authors

All authors have made a substantial contribution to the manuscript and the research presented.

## Conflict of Interest Statement

None declared.
